# Non-specific phospholipase C4 mediates response to aluminum toxicity in *Arabidopsis thaliana*

**DOI:** 10.3389/fpls.2015.00066

**Published:** 2015-02-16

**Authors:** Přemysl Pejchar, Martin Potocký, Zuzana Krčková, Jitka Brouzdová, Michal Daněk, Jan Martinec

**Affiliations:** Institute of Experimental Botany, Academy of Sciences of the Czech Republic, PragueCzech Republic

**Keywords:** aluminum toxicity, *Arabidopsis*, diacylglycerol, non-specific phospholipase C, plasma membrane, pollen tube, signaling, tobacco

## Abstract

Aluminum ions (Al) have been recognized as a major toxic factor for crop production in acidic soils. The first indication of the Al toxicity in plants is the cessation of root growth, but the mechanism of root growth inhibition is largely unknown. Here we examined the impact of Al on the expression, activity, and function of the non-specific phospholipase C4 (NPC4), a plasma membrane-bound isoform of NPC, a member of the plant phospholipase family, in *Arabidopsis thaliana*. We observed a lower expression of *NPC4* using β-glucuronidase assay and a decreased formation of labeled diacylglycerol, product of NPC activity, using fluorescently labeled phosphatidylcholine as a phospholipase substrate in *Arabidopsis* WT seedlings treated with AlCl_3_ for 2 h. The effect on *in situ* NPC activity persisted for longer Al treatment periods (8, 14 h). Interestingly, in seedlings overexpressing *NPC4*, the Al-mediated NPC-inhibiting effect was alleviated at 14 h. However, *in vitro* activity and localization of NPC4 were not affected by Al, thus excluding direct inhibition by Al ions or possible translocation of NPC4 as the mechanisms involved in NPC-inhibiting effect. Furthermore, the growth of tobacco pollen tubes rapidly arrested by Al was partially rescued by the overexpression of *AtNPC4* while *Arabidopsis npc4* knockout lines were found to be more sensitive to Al stress during long-term exposure of Al at low phosphate conditions. Our observations suggest that NPC4 plays a role in both early and long-term responses to Al stress.

## INTRODUCTION

Aluminum (Al) toxicity represents a major growth-limiting factor for the regions with acid soils. Low pH of soil enables the release of toxic Al ions from its insoluble forms fixed in soil minerals. Prolonged exposure to Al ions leads to changes in root morphology, e.g., root thickening, bursting, changes in the cell wall architecture, and even cell death. However, the first indication of the Al toxicity in plants is rapid cessation of root growth. The root tip has been found to be the most Al-responsive part of roots (Panda et al., 2009). Although molecular mechanisms of the prompt Al-mediated root growth inhibition are largely unclear, research on the targets of Al action in plants has demonstrated that Al enters and binds to the apoplast (Wissemeier and Horst, 1995) and changes the properties of the PM. A number of physiologically important processes connected with PM are affected by Al. Well documented early consequences of Al toxicity are lipid peroxidation (Boscolo et al., 2003), the disruption of ion fluxes (Matsumoto, 2000), the disruption of calcium homeostasis (Rengel and Zhang, 2003), the inhibition of nitric oxide synthase (Tian et al., 2007), effects on the cytoskeleton (Sivaguru et al., 1999, 2003; Schwarzerová et al., 2002), and the depolarization of the PM (Sivaguru et al., 2003; Illéš et al., 2006). It has been found that rapid Al-mediated inhibition of root growth is related to the loss of PM fluidity and the inhibition of endocytosis (Illéš et al., 2006; Krtková et al., 2012) and it is controlled through local auxin biosynthesis and signaling (Shen et al., 2008; Yang et al., 2014). The rapid response of root growth suggests that signaling pathways are a part of the mechanism participating in Al toxicity.

Phospholipid-signaling pathway is now considered to be one of the important plant signaling mechanisms involved in many different reactions of plants to environmental factors such as drought, cold, salinity, or pathogen attack [for review see Munnik (2010) and Wang (2014)]. Al has been shown to affect the phospholipid-signaling pathway as well. Changes of phospholipase A_2_ activity *in vitro* (Jones and Kochian, 1997), PLD activity (Pejchar et al., 2008; Zhao et al., 2011), and PI-PLC activity (Jones and Kochian, 1995; Martínez-Estévez et al., 2003; Ramos-Díaz et al., 2007) after Al treatment were demonstrated. In addition to PI-PLC, NPC, an enzyme that is able to hydrolyze phosphatidylcholine (PC) instead of PIP_2_, was characterized in plants (Nakamura et al., 2005) in relation with phospholipid-to-galactosyl DAG exchange (Andersson et al., 2005; Nakamura et al., 2005; Gaude et al., 2008; Tjellström et al., 2008), elicitor signaling (Scherer et al., 2002), root development (Wimalasekera et al., 2010), hormone signaling (Peters et al., 2010; Wimalasekera et al., 2010), salt stress (Kocourková et al., 2011; Peters et al., 2014), and Al stress (Pejchar et al., 2010).

*Arabidopsis* NPC gene family consists of six members, denoted *NPC1-NPC6*, exhibiting differences in their localization and in their biochemical properties [for review see Pokotylo et al. (2013)]. Briefly, experimentally non-characterized NPC1, NPC2, and NPC6 were supposed to contain putative N-terminal signal peptide with predicted localization in endomembranes and specific organelles (Pokotylo et al., 2013). NPC3 was described to lack the ability to hydrolyze PC (Reddy et al., 2010), NPC4 to be PM-bound protein (Nakamura et al., 2005), NPC5 to be cytosolic-localized enzyme expressed only in floral organs under normal conditions (Gaude et al., 2008; Pokotylo et al., 2013). NPC4 and NPC5 were able to hydrolyze PC, however, NPC5 possessed 40-fold lower hydrolytic activity than NPC4 (Gaude et al., 2008).

We previously demonstrated that Al ions inhibit the formation of DAG generated by NPC in tobacco BY-2 cell line and pollen tubes, inhibit the growth of tobacco pollen tubes and that this growth, arrested by Al, can be rescued by an externally added DAG (Pejchar et al., 2010). This raises the following question: which NPC isoform is Al-targeted and what is the role of DAG in aluminum toxicity?

Here we report our findings that Al ions inhibit the expression of *NPC4* and decrease its enzymatic activity. However, the latter effect is caused neither by the direct NPC4 inhibition by Al ions nor by NPC4 translocation. Moreover, the overexpression of *AtNPC4* rapidly alleviated Al-mediated retardation of tobacco pollen tubes while *Arabidopsis npc4* knockout lines were found to be more sensitive to Al stress during long-term exposure of Al at low phosphate (P) conditions.

## MATERIALS AND METHODS

### PLANT MATERIAL

*Arabidopsis thaliana* Columbia (Col-0) seeds were obtained from Lehle seeds and used as wild-type (WT) controls. The T-DNA insertion line *npc4* (SALK_046713) used in our experiments was characterized earlier (Wimalasekera et al., 2010). *Arabidopsis* plants were grown on agar plates containing 2.2 gl^-1^ 1/2 MS basal salts and 1% (w/v) agar (pH 5.8). Seeds were surface sterilized with 30% (v/v) bleach solution for 10 min and rinsed five times with sterile water. To synchronize seed germination, the agar plates were kept for 3 days in a dark at 4°C. The plants were grown in the vertical position in a growth chamber at 22°C under long day conditions (16/8 h light/dark cycle). Tobacco (*Nicotiana tabacum* cv. Samsun) pollen grains germinated on simple sucrose medium (Pleskot et al., 2012) containing 10% (w/v) sucrose and 0.01% (w/v) boric acid solidified by 0.5% (w/v) agar were used for biolistic transformation.

### ASSAYING NON-SPECIFIC PHOSPHOLIPASE C ACTIVITY *IN SITU* AND *IN VITRO*

The NPC activity in *Arabidopsis* seedlings was measured according to Kocourková et al. (2011). Seven-day-old *Arabidopsis* seedlings (five seedlings for each sample) were transferred from liquid MS solution to 1/8 MS medium containing 10 μM AlCl_3_, pH 4 and labeled with 0.66 μg ml^-1^ of fluorescent PC (BODIPY-PC, D-3771, Invitrogen, USA). Seedlings were incubated on an orbital shaker at 23°C for 2 h. NPC activity *in vitro* was measured according to Pejchar et al. (2013) using β-BODIPY-PC (D-3792, Invitrogen, USA). The identification of a BODIPY-DAG corresponding spot was based on a comparison with the BODIPY-DAG standard prepared as described earlier (Pejchar et al., 2010).

### HISTOCHEMICAL β-GLUCURONIDASE STAINING

The construction of promoter:GUS plants was described previously (Wimalasekera et al., 2010). Seeds of *pNPC4:GUS* were grown on agar plates under the same conditions as described in Section “Plant Material.” Ten-day-old seedlings were transferred to a 24-well plate containing 1 ml of 1/8 MS solution with or without 100 μM AlCl_3_, pH 4 for 24 h. The histochemical GUS assay (Jefferson et al., 1987) was carried out according to Kocourková et al. (2011).

### MOLECULAR CLONING, TRANSFORMATIONS, ISOLATION

***His:AtNPC4.*** The AtNPC4 coding sequence was amplified from *Arabidopsis* Col-0 cDNA with the specific forward primer 5^′^-CGCGAATTCATGATCGAGACGACCAAA-3^′^ and the reverse primer 5^′^-GCCCTCGAGTCAATCATGGCGAATAAAG-3^′^ by PCR using Phusion DNA polymerase (Finnzymes), digested with XhoI and EcoRI enzymes and cloned into the pET30a(+) vector (Novagen). The expression vector was transformed into the *Escherichia coli* strain BL21 and cells were grown overnight at 37°C. After subculturing into fresh medium, the cells were grown at 16°C to an OD_600_ of approximately 0.4, then induced overnight with 0.1 mM isopropyl thio-β-D-galactoside. The cells were harvested by centrifugation (5000 ×*g*, 10 min), resuspended in an assay buffer (50 mM Tris-HCl; pH 7.3, 50 mM NaCl, 5% glycerol; Nakamura et al., 2005), and sonicated after 10 min treatment with lysozyme (1 mg ml^-1^). The lysed cell suspension was centrifuged (10000 ×*g*, 10 min) and supernatant was used for an enzyme activity assay. The western blot analysis was performed under reducing and denaturation conditions with SDS electrophoresis. 6x His tag was detected with Anti-His HRP Conjugate (Qiagen).

***35S::AtNPC4/35S::GFP:AtNPC4.*** AtNPC4 cloned into the pENTR223.1 entry vector (Gateway^TM^ clone G12733, *Arabidopsis* Biological Resource Center) was recombined by LR reaction into the Gateway binary vector pGWB2 (*35S::AtNPC4*) or pGWB6 (*35S::GFP:AtNPC4*) under the control of the CaMV 35S promoter (Nakagawa et al., 2007). Constructs were transferred into *Agrobacterium tumefaciens* strain GV2260 and used to transform *Arabidopsis* Col-0 WT plants by the floral dip method (Clough and Bent, 1998). Transformants were selected on agar plates containing 50 μg ml^-1^ kanamycin and 50 μg ml^-1^ hygromycin B. Expression levels of *NPC4* in 10-day-old T3 seedlings of homozygous lines were measured using the quantitative RT-PCR. Lines with the highest expression levels of *NPC4* were used in experiments.

***Lat52::AtNPC4:YFP.*** The AtNPC4 coding sequence flanked by NgoMIV and ApaI sites was generated by PCR with Phusion DNA polymerase (Finnzymes) using the specific forward primer 5^′^-ATAGCCGGCATGATCGAGACGACCAA-3^′^ and the reverse primer 5^′^-TATGGGCCCATCATGGCGAATAAAGCA-3^′^. An amplified product was introduced into the multiple cloning sites of the pollen expression vector pHD32. The pHD32 vector (Lat52::MCS::GA5::YFP::NOS; Klahre et al., 2006) was kindly provided by Prof. Benedikt Kost (University of Erlangen-Nuremberg, Erlangen, Germany). This construct allowed the pollen-specific expression and visualization of AtNPC4 protein fusions controlled by the *Lat52* promoter (Twell et al., 1991). The expression vector was transferred into tobacco pollen grains germinating on solid culture medium by particle bombardment using a helium-driven particle delivery system (PDS-1000/He; Bio-Rad, Hercules, CA, USA) as previously described (Kost et al., 1998). Particles were coated with 1 μg DNA.

### MICROSCOPY

For live-cell imaging, a Zeiss LSM 5 DUO confocal laser scanning microscope with a 940 Zeiss C-Apochromat 340/1.2 water-corrected objective was used. For YFP/GFP imaging, singletrack acquisitions with 514 nm excitation, a 458/514 nm dichroic mirror, a 530–600 nm emission filter (YFP) and 488 nm excitation, a 488 nm dichroic mirror, a 505–550 emission filter (GFP) were used.

### EVALUATION OF AL EFFECT

To analyze root length, five-day-old seedlings grown on agar (for details see Plant Material) were transferred on agar plates containing 1/8 MS, pH 4 supplemented with 200 μM AlCl_3_. After 9 day incubation, plates were scanned (Canon CanoScan 8800F) and the root growth was measured using the JMicroVision 1.2.7 software.

To measure pollen tubes length, tobacco pollen was transiently transformed with *AtNPC4:YFP* by particle bombardment. After 6 h of germination in dark, pollen tubes were incubated in liquid simple sucrose medium (pH 5) with or without 50 μM AlCl_3_ for additional 2 h. Mean growth rate of pollen tubes expressing *AtNPC4:YFP* and vector-only control was evaluated using the fluorescence microscope Olympus BX-51.

To determine survival rate, 7-day-old seedlings grown on agar (for details see Plant Material) were transferred to 6-well plates with liquid 1/8 Hoagland’s (Kocourková et al., 2011) solution (pH 4) with 100 μM AlCl_3_ for 22 days. Plates pictures were taken by Nikon SMZ 1500 zoom stereoscopic microscope coupled to a Nikon DS-5M digital camera. The survival rate was calculated as a number of viable true leaves.

## RESULTS

Aluminum ions were described to inhibit the formation of DAG generated by NPC in tobacco cell line BY-2 and in tobacco pollen tubes (Pejchar et al., 2010). In order to find an NPC isoform that is responsible for the decrease of DAG formation during Al stress, described biochemical properties and localizations of all NPC isoforms were taken into account (see Introduction for details). Altogether, NPC4 was the first candidate to be investigated.

### EXPRESSION OF *NPC4* IN ROOT TIPS IS DECREASED DURING AL STRESS

**FIGURE 1:**
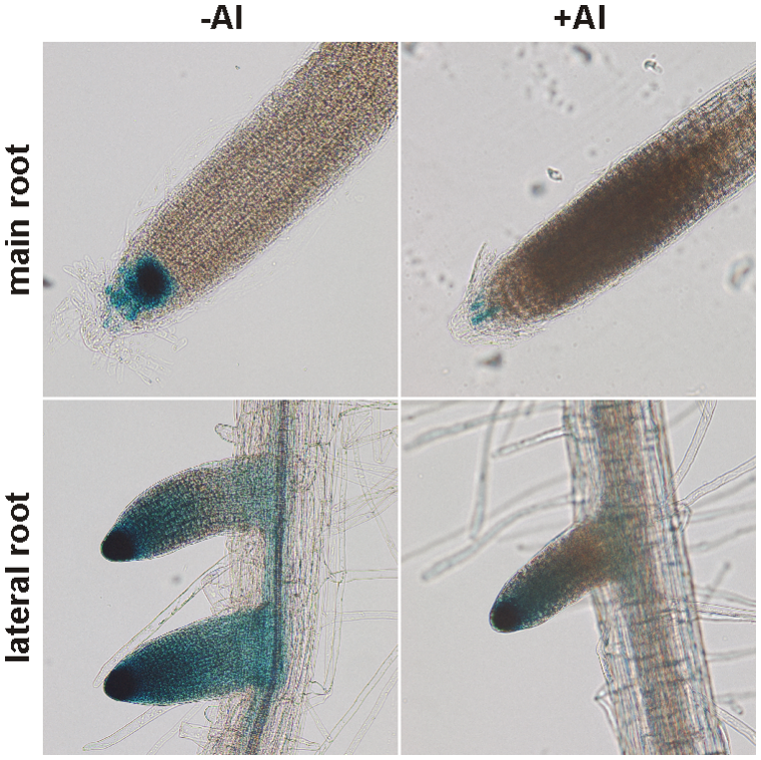
**Histochemical analysis of *pNPC4:GUS* expression in Al-treated *Arabidopsis* seedlings.** Ten-day-old *Arabidopsis* seedlings grown on agar were transferred to liquid 1/8 MS solution with or without AlCl_3_ for 24 h. Final observations were done on a zoom stereoscopic microscope. This experiment was repeated twice with similar results.

Considering all available data about the NPC gene family and given that the root and PM are the main targets of Al toxicity, we chose NPC4 to study its role in Al stress. Although *NPC4* is not the most abundant *NPC* gene expressed in roots (Peters et al., 2010; Wimalasekera et al., 2010; Pokotylo et al., 2013) it was described as the isoform with the strongest response to abiotic stress in plants (Kocourková et al., 2011). In our previous studies, we have shown that the expression of *NPC4*, investigated using *pNPC4:GUS* plants, was largely localized in the root tip (Wimalasekera et al., 2010; Kocourková et al., 2011). In this study, we performed histochemical analysis of *Arabidopsis pNPC4:GUS* seedlings treated with AlCl_3_ to observe changes in the expression pattern of *NPC4* during Al stress. GUS staining in both control and Al-treated seedlings was found in the apical meristem and partly in the elongation zone of main and lateral root but the intensity of GUS staining signal was lower in Al-treated seedlings (**Figure 1**). These observations suggest that the *NPC4* expression is decreased during Al stress in *Arabidopsis* seedlings.

### AL-INDUCED INHIBITION OF DIACYLGLYCEROL FORMATION IS ALLEVIATED IN *NPC4*-OVEREXPRESSING SEEDLINGS

**FIGURE 2:**
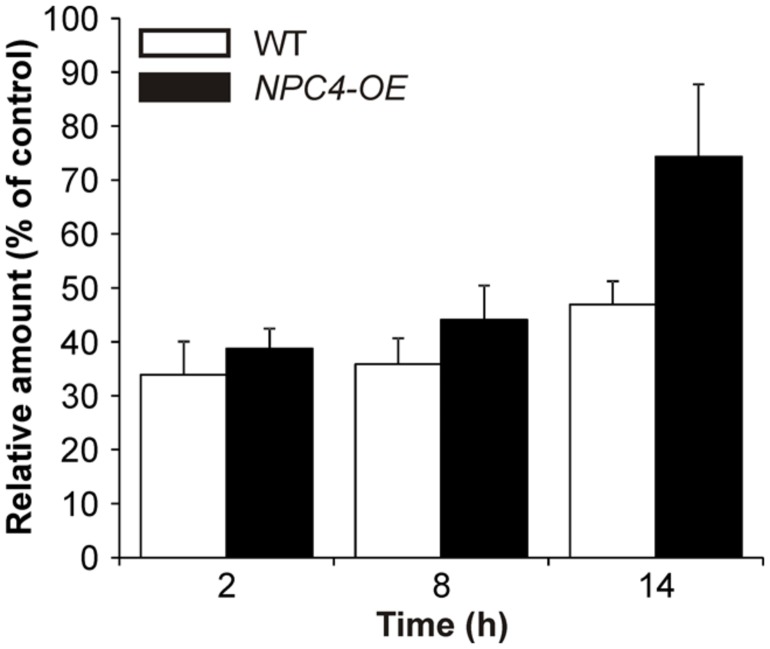
**BODIPY-diacylglycerol (DAG) production in *Arabidopsis* seedlings treated with Al for different times.** Seven-day-old WT and *NPC4*-overexpressing seedlings were treated with 10 μM AlCl_3_ for different time intervals (0, 6, and 12 h) and then incubated with BODIPY-phosphatidylcholine (PC) for 2 h. Lipids were extracted at the time intervals indicated, separated by high-performance thin layer chromatography and quantified. Each value is related to the control non-treated cells (100%). The plotted values are the means + SEM from three independent experiments with parallel samples. NPC, non-specific phospholipase C.

The involvement of NPC4 in Al stress was also examined in the level of its activity. Because we used a different plant model organism than in our previous work (Pejchar et al., 2010), we first tested the reaction of *Arabidopsis* seedlings to Al stress. To study changes in the DAG pattern under Al stress, we used the fluorescent derivative of PC (BODIPY-PC) as a phospholipase substrate. When seven-day-old seedlings were treated with different concentrations of AlCl_3_ in the presence of BODIPY-PC for 2 h, a concentration-dependent inhibiting effect of Al on BODIPY-DAG formation was observed (Pejchar, unpublished), revealing 10 μM AlCl_3_ as a working concentration for *in situ* activity measurement (**Figure 2**).

Consequently, we tested the hypothesis that NPC4 is also the targeted isoform at activity level during Al stress and it is responsible for the inhibition of DAG formation. Therefore, a stable *Arabidopsis* line overexpressing *NPC4* under the control of *35S* promoter (*NPC4-OE*) was prepared and was monitored to BODIPY-DAG formation after Al treatment and compared to Al-treated WT seedlings. First, our HP-TLC analysis of the labeled products showed that the trend of the Al-induced BODIPY-DAG inhibition (∼35% of control, non-treated seedlings) in WT seedlings was similar also for prolonged treatments with 10 μM AlCl_3_ (**Figure 2**) with a slightly diminished effect of Al after 14 h of treatment (∼47% of control). *NPC4-OE* seedlings were slightly less sensitive to Al compared to WT when treated for 2 and 8 h. Intriguingly, the overexpression of *NPC4* resulted in a more pronounced difference (∼74% compared to ∼47% of control) after 14 h Al treatment. This suggests that NPC4 is an Al-sensitive NPC isoform on activity level during Al stress, as well.

### THE EFFECT OF AL ON NPC4 IS NEITHER DUE TO DIRECT INHIBITION OF NPC4 ENZYME NOR DUE TO NPC4 TRANSLOCATION

**FIGURE 3:**
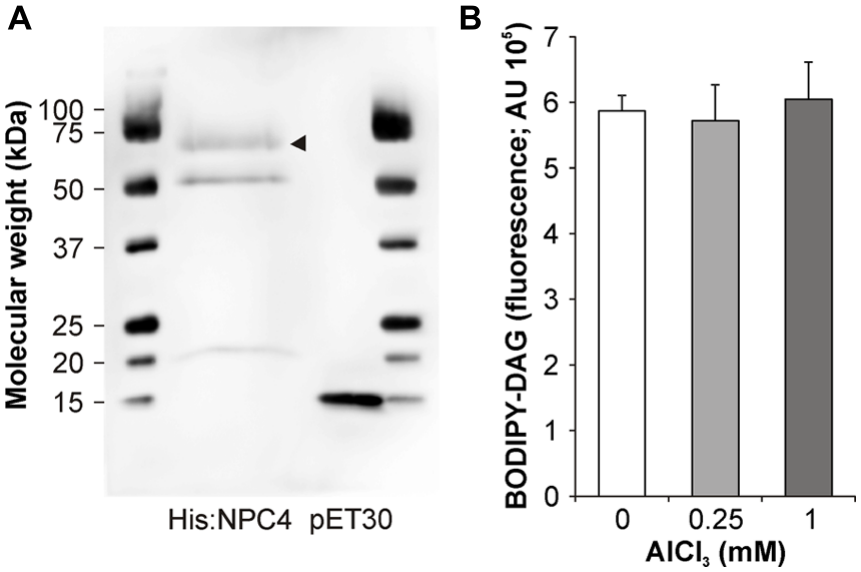
***In vitro* activity of NPC4 is not altered by Al.** **(A)** Western blot of His:NPC4 and vector control was probed with Anti-His HRP Conjugate (Qiagen). Arrow head indicates His:NPC4 protein. **(B)** NPC activity was determined *in vitro* using fluorescent substrate β-BODIPY-PC in the presence of AlCl_3_. The plotted values are the means + SD from two independent experiments performed in duplicates (*n* = 4). DAG, diacylglycerol.

To test possible mechanisms that influenced NPC4 activity in Al stress, a heterologously expressed NPC4 protein was prepared (**Figure 3A**) and incubated with Al *in vitro* to detect possible direct inhibition of NPC4 by Al. However, β-BODIPY-DAG formation in Al-treated samples was not affected compared to non-treated samples (**Figure 3B**) indicating that NPC4 is not directly inhibited by Al.

**FIGURE 4:**
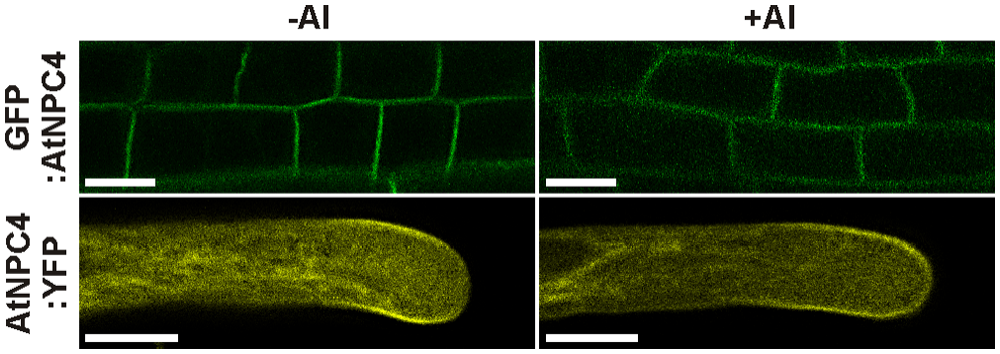
**Localization of NPC4 is not changed by Al treatment.** Influence of AlCl_3_ on localization of AtNPC4 was observed in 7-day-old stable *Arabidopsis* transformants (GFP:AtNPC4, upper panels) and transiently transformed tobacco pollen tubes (AtNPC4:YFP, lower panels) by confocal laser scanning microscopy. Bars, 10 μm. NPC, non-specific phospholipase C.

Given that PM is well-documented cellular target of Al and that NPC4 was described as a PM-localized protein (Nakamura et al., 2005), we studied possible translocation of NPC4 from PM during Al treatment, which should cause a decrease in the DAG formation. The protein translocation under stress conditions was previously described in plants for another phospholipase type, PLD (Wang et al., 2000; Bargmann et al., 2006). To check this mechanism, stable *Arabidopsis* transformants harboring fusion protein GFP:NPC4 were prepared. Seven-day-old seedlings were transferred to 1% (w/v) sucrose (pH 4.3) solution containing 50 μM AlCl_3_ and the localization of GFP:NPC4 in roots was investigated with a laser scanning confocal microscope. In control, non-treated seedlings, the PM localization of GFP:NPC4 was detected confirming previously published results (Nakamura et al., 2005). The localization of GFP:NPC4 remained unchanged in Al-treated seedlings (**Figure 4**, upper panels). The same results were obtained for transiently transformed tobacco pollen tubes expressing *AtNPC4:YFP* under the control of pollen specific *Lat52* promoter (**Figure 4**, lower panels), another plant model organism used in this study. These results provide evidence that NPC4 translocation is not a mechanism that induces DAG decrease during Al stress.

### OVEREXPRESSION OF *AtNPC4* PARTIALLY RESTORED GROWTH OF TOBACCO POLLEN TUBES UNDER AL STRESS

**FIGURE 5:**
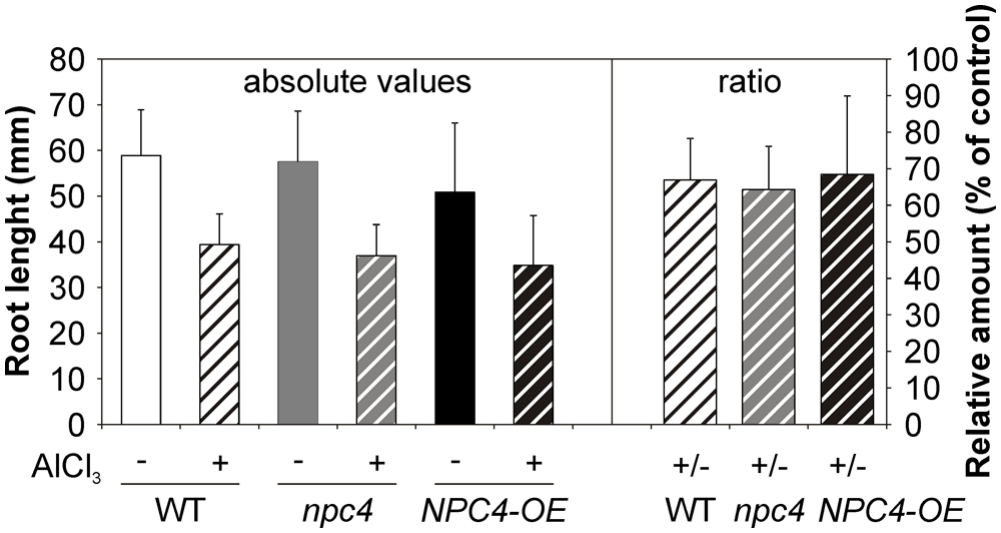
**Root growth assay of WT, *npc4* and *NPC4*-overexpressing *Arabidopsis* seedlings treated with Al.** Five-day-old seedlings were transferred on agar plates containing 1/8 MS supplemented with AlCl_3_. After 9 day incubation, the root growth was measured and compared to non-treated controls. The plotted values represent the means (left panel) and ratios of Al-treated/non-treated control seedlings (right panel) + SD from two independent experiments. At least 16 seedlings were measured for each variant. NPC, non-specific phospholipase C.

Next, a root growth phenotype under Al stress was investigated in *Arabidopsis* WT, *npc4* knockout line and *NPC4-OE*. Five-day-old seedlings grown on agar MS medium were transferred on agar 1/8 MS medium containing 200 μM AlCl_3_. The growth of the main root of all lines tested was retarded in the presence of Al. However, the root growth ratio between Al-treated and non-treated seedlings was not different for examined lines (**Figure 5**).

**FIGURE 6:**
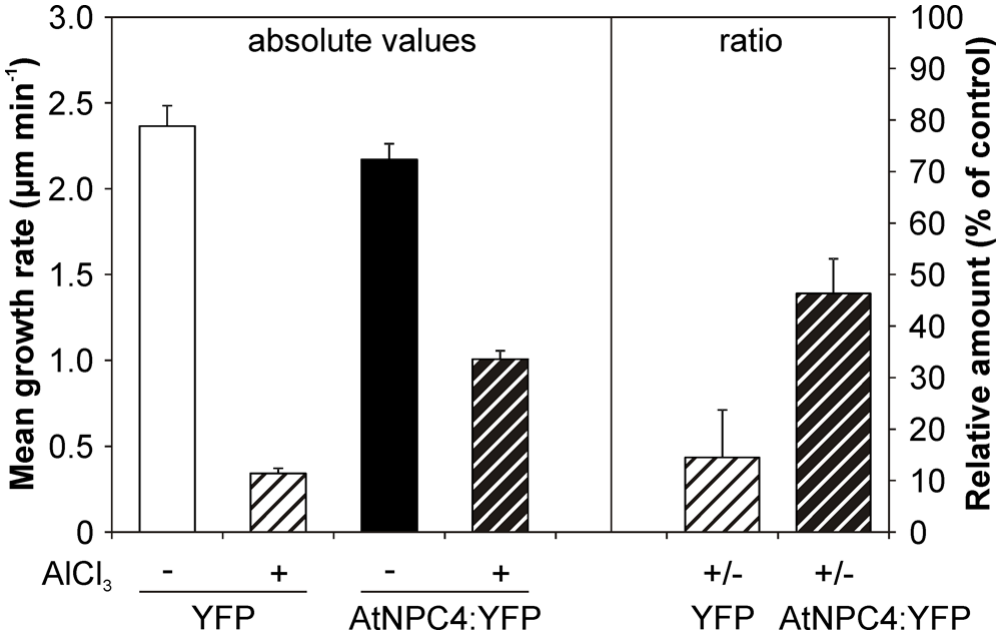
**Effect of Al on the growth rate of tobacco pollen tubes transiently expressing *AtNPC4:YFP*.** Tobacco pollen was sown on solid germination medium and transiently transformed with *AtNPC4:YFP* by particle bombardment. After 6 h of germination, pollen tubes were incubated with 50 μM AlCl_3_ for additional 2 h. Mean growth rate of pollen tubes expressing *AtNPC4:YFP* or vector-only control was evaluated using a fluorescence microscope. Data shown are from two independent experiments and represent means + SEM. At least 21 pollen tubes were measured for each variant. NPC, non-specific phospholipase C.

This could be explained by possible compensation of NPC4 function in stable transformants by another member of lipid signaling enzyme network. To bypass this, we employed transient transformation in heterologous system of tobacco pollen. Moreover, in our previously published study (Pejchar et al., 2010), DAG was shown to restore growth inhibition caused by Al treatment in tobacco pollen tubes. To test the possible role of NPC4 in this DAG function, tobacco pollen tubes were transiently transformed with *AtNPC4:YFP* under the control of pollen specific *Lat52* promoter and the length of pollen tubes overexpressing *AtNPC4:YFP* was determined in the presence of Al. In the control pollen tubes overexpressing *YFP* alone, the cytoplasmic YFP localization was found (data not shown) and the mean growth rate was 2.36 ± 0.12 μm min^-1^ (**Figure 6**). Al-treatment caused the inhibition of control pollen tubes growth to approximately 15% (0.34 ± 0.03 μm min^-1^) of non-treated cells. Control pollen tubes overexpressing *AtNPC4:YFP* showed a slightly decreased mean growth rate (2.17 ± 0.09 μm min^-1^) compared to *YFP* only. However, in the presence of Al the mean growth of pollen tubes overexpressing *AtNPC4:YFP* (1.01 ± 0.05 μm min^-1^) was higher compared to Al-treated vector-only control (**Figure 6**). Taken together, these results clearly demonstrate that the role of DAG as a growth activator in Al stress is mediated by NPC4 activity.

### *Arabidopsis npc4* SEEDLINGS ARE MORE SENSITIVE TO AL STRESS IN PHOSPHATE DEFICIENCY

**FIGURE 7:**
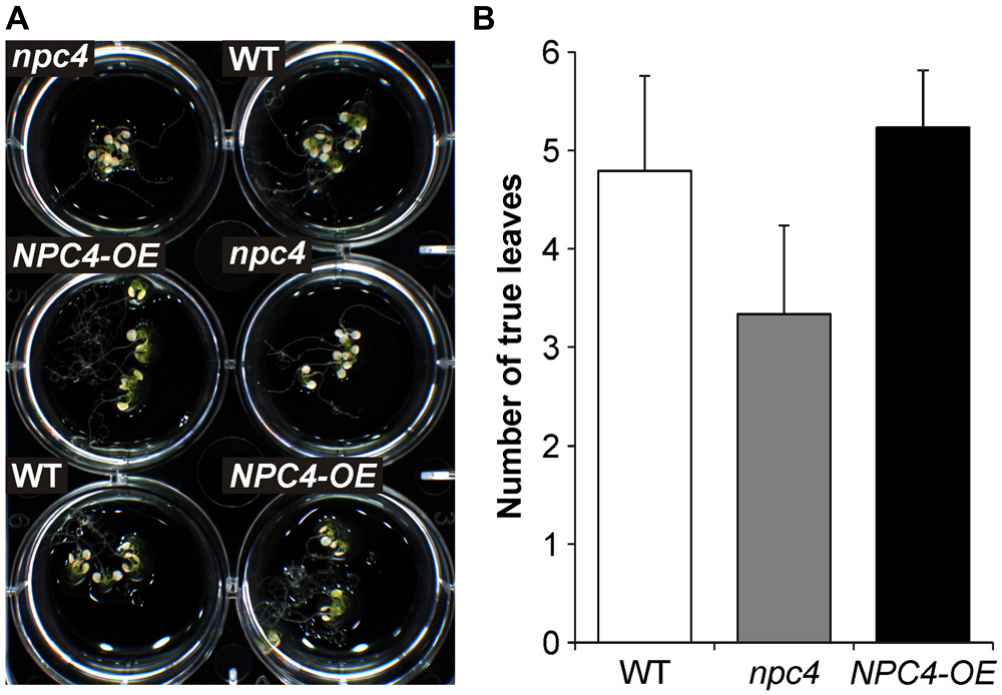
**Effect of Al on the survivance of WT, *npc4,* and *NPC4*-overexpressing *Arabidopsis* seedlings under phosphate deficiency.** **(A)** Seven-day-old seedlings grown on agar were transferred to liquid 1/8 Hoagland’s solution with AlCl_3_ for 22 days. **(B)** Quantitative analysis of survival rate calculated as a number of viable true leaves. The plotted values are the means + SD from two independent experiments (*n* = 50). NPC, non-specific phospholipase C.

Based on the results concerning the role of NPC4 activity in longer time period of Al treatment (**Figure 2**), a long-term survival experiment was also performed. Seven-day-old seedlings grown on agar were transferred to liquid 1/8 Hoagland’s solution with AlCl_3_ for 22 days. However, all tested lines (WT, *npc4*, *NPC4-OE*) showed no difference in their survival rate (data not shown). Since NPC4 play a role in P starvation (Nakamura et al., 2005) and aluminum stress and P deficiency co-exist in acid soils (Ruíz-Herrera and López-Bucio, 2013), the same experiment was repeated under P deficiency conditions. Differences in both root abundance (**Figure 7A**) and survival rate (**Figure 7A**) were found between WT and *npc4* seedlings after Al treatment. Seedlings of *npc4* were able to form only a weaker root system and significantly (*t*-test, *p* < 0.0001) less true leaves comparing to WT. This suggests that *npc4* seedlings are more sensitive to Al stress at low P conditions. In contrast to this finding, we also saw that *NPC4-OE* seedlings formed a more abundant root system compared to WT (**Figure 7A**). However, only a minimal increase of survival rate was found for *NPC4-OE* (**Figures 7A,B**). Collectively, these data strongly support the involvement of NPC4 in response to long-term Al exposure.

## DISCUSSION

Several physiologically important cellular processes are affected by Al, the major growth-limiting factor for the regions with acid soils. However, time sequence and the exact mechanism of processes involved in Al stress are still under investigation. Phospholipases, namely PI-PLC and PLD, have been shown to be affected within minutes as well as in longer time periods after Al treatment (Martínez-Estévez et al., 2003; Ramos-Díaz et al., 2007; Pejchar et al., 2008; Zhao et al., 2011). We previously described that the formation of DAG generated by NPC is rapidly inhibited by Al in tobacco cell line BY-2 and in tobacco pollen tubes and that Al inhibits growth of tobacco pollen tubes. These results, together with the fact that Al-mediated growth arrest can be rescued by an externally added DAG (Pejchar et al., 2010), raised a question which NPC isoform is Al-targeted in aluminum toxicity. Here we showed that NPC isoform NPC4 is involved in the response of *A. thaliana* to Al exposure.

We selected *Arabidopsis* NPC4 as the primary candidate gene based on the expression and localization criteria (see above). The expression analysis using *pNPC4:GUS* assay showed that the localization and the intensity of *NPC4* expression in the non-treated main root was the same as published previously (Wimalasekera et al., 2010). However, we found stronger GUS staining in non-treated lateral roots (**Figure 1**). This difference could be explained by different experimental conditions used as a control for Al treatments (pH 4) or by small variation of seedling age used for GUS assay. More importantly, Al treatment caused reduction in GUS staining in both main and lateral roots suggesting that the *NPC4* expression is decreased during Al treatment. In addition, *pNPC4:GUS* expression was confined mainly to the root tip (**Figure 1**), a plant tissue that was found to be the most Al-responsive part of roots (Panda et al., 2009).

Although we confirmed the generally accepted symptom of Al toxicity, root growth inhibition, and we observed diminished *NPC4* expression after Al treatment, we were not able to determine differences between Al-treated WT, *npc4*, and *NPC4-OE Arabidopsis* seedlings (**Figure 5**). We have two hypotheses regarding the lack of the differences. First, it is possible that no differences were found because NPC4 is involved in other aspects of the response to Al stress than in the studied root growth phenotype. Alternatively, the lack of the differences is caused by the positive or negative compensatory effect of another NPC isoform (or other lipid signaling genes) that may be up- or down-regulated in *npc4* knockout or stable *NPC4-OE* line, respectively. Similar observations were indeed described for studies dealing with other lipid signaling genes, such as PLD (Bargmann et al., 2009; Johansson et al., 2014) or phospholipase A (Rietz et al., 2010). All six NPC sequences are highly conserved with four invariable motives, however, the C-termini form the most divergent part of NPC sequences, with distinct lengths and sequence conservation among NPC subfamilies. This may be the part of the molecule responsible for the functional differences of various NPC isoforms through facilitating interactions with other proteins or defining protein localization (Pokotylo et al., 2013). Interestingly, while NPC3-5 are found in triplicate in *Arabidopsis* genome and members of this subfamily can also be found in other monocot and dicot species, it seems to be missing in gymnosperms and it is also absent in *Solanaceae* (Potocký, unpublished). Taking advantage of this, we employed a strategy of studying the effect of Al in tobacco pollen tubes heterologously overexpressing *AtNPC4:YFP* under the control of strong pollen specific *Lat52* promoter. The growth of tobacco pollen tubes was rapidly arrested by Al, supporting our previous results (Pejchar et al., 2010), and was partially rescued by the overexpression of *AtNPC4:YFP* (**Figure 6**). Together with Al-mediated pollen growth inhibition rescue by exogenously added DAG (Pejchar et al., 2010), this strongly suggests that NPC4-generated DAG plays a role in the response to the Al-mediated toxicity.

Because a different plant model organism than in our previous work was used, we next tested the reaction of *Arabidopsis* seedlings to Al stress in the view of NPC activity. We utilized the same fluorescent derivative of PC as a phospholipase substrate and found a similar Al-mediated NPC-inhibiting effect (**Figure 2**) as for the tobacco cell line BY-2 and pollen tubes (Pejchar et al., 2010) suggesting that this phenomenon could be conserved across the plant kingdom. Interestingly, effects on *pNPC4:GUS* expression and NPC activity in Al-treated plants were in the opposite way than described for another abiotic stress that targets root, salt treatment (Kocourková et al., 2011). To test the hypothesis whether NPC4 is responsible for inhibition of DAG formation during Al stress, we prepared the stable *Arabidopsis* lines overexpressing *NPC4* and we compared the ratio of NPC activity in Al-treated/non-treated seedlings to WT seedlings. The differences between WT and *NPC4-OE* seedlings were slowly pronounced in time with the most evident change obtained for the seedlings treated with Al for 14 h (**Figure 2**) indicating that NPC4 activity is altered by Al gradually. Two possible mechanisms that could be responsible for the decrease of NPC activity were examined. The inhibition of phospholipase activity *in vitro* in cellular fractions is well documented for different toxic metals (Pokotylo et al., 2014) and for Al as well (Martínez-Estévez et al., 2003; Pejchar et al., 2008). Here, the direct inhibition of heterologously expressed NPC4 enzyme by Al was tested with no alteration in activity detected even for high AlCl_3_ concentrations (**Figure 3**). The second possible mechanism, enzyme translocation, was previously described in plants under stress conditions for another phospholipase type, PLD (Wang et al., 2000; Bargmann et al., 2006). However, Al treatment had no effect on NPC4 localization neither in *Arabidopsis* seedlings nor tobacco pollen tubes (**Figure 4**).

Moreover, AtNPC4:YFP was found on PM in subapical region of growing pollen tube (**Figure 4**) and thus partially co-localize with Cys1:YFP that was used as a DAG marker in tobacco pollen tubes (Potocký et al., 2014). DAG is an important signaling phospholipid in animals but its signaling role in plant cells is still under debate. Meijer and Munnik (2003) reported that DAG as a product of PIP_2_ hydrolysis is rapidly phosphorylated by DAG kinase to PA, which plays an active role in the plant signaling processes. However, a number of studies imply that DAG is likely to act as a signaling molecule in some plant systems including *Arabidopsis* seedlings and tobacco pollen tubes [reviewed in Dong et al. (2012)]. DAG is also known to be important in the structure and dynamics of biological membranes, where it can influence membrane curvature and induce unstable, asymmetric regions in membrane bilayers important for membrane fusion processes (Carrasco and Mérida, 2007; Haucke and Di Paolo, 2007), events that occur in many physiological processes, such as exocytosis, endocytosis, membrane biogenesis, and cell division. Moreover Al-mediated inhibition of root growth was found to be connected with the inhibition of endocytosis (Illéš et al., 2006; Krtková et al., 2012) and controlled through local auxin biosynthesis and signaling (Shen et al., 2008; Yang et al., 2014). Not incurious, expression of *NPC4* was increased and *npc4* seedlings exhibit shorter primary root and smaller density of lateral roots after auxin treatment (Wimalasekera et al., 2010). Thus, it is worthwhile to note that the inhibition of DAG formation during Al stress might affect the mentioned processes and rapidly inhibit growth. On the other hand, our long-term experiment revealed that *npc4* seedlings were more sensitive to Al stress while *NPC4-OE* seedlings formed a more abundant root system (**Figure 7**). This suggests that NPC4/DAG functions differently, more likely participating in lipid turnover and membrane remodeling, respectively.

In summary, our results suggest that the previously described involvement of NPC in response to Al stress is mediated by NPC4 in *A. thaliana*.

## Conflict of Interest Statement

The authors declare that the research was conducted in the absence of any commercial or financial relationships that could be construed as a potential conflict of interest.

## ABBREVIATIONS

BODIPY: 4,4-difluoro-4-bora-3a,4a-diaza-s-indacene
BY-2: bright yellow 2
DAG: diacylglycerol
GUS: β-glucuronidase
HP-TLC: high-performance thin-layer chromatography
MS: Murashige-Skoog
NPC: non-specific phospholipase C
PA: phosphatidic acid
PIP2: phosphatidylinositol 4,5-bisphosphate
PI-PLC: phosphatidylinositol-specific phospholipase C
PLD: phospholipase D
PM: plasma membrane
